# Mixed Adenocarcinoma Plus Undifferentiated Carcinoma of the Descending Colon Presenting as a Bulky Retroperitoneal Mass

**DOI:** 10.1002/kjm2.70320

**Published:** 2026-11-14

**Authors:** Pin‐Yuan Chen, Peir‐In Liang, Jaw‐Yuan Wang, Hsiang‐Lin Tsai

**Affiliations:** ^1^ Division of Colorectal Surgery, Department of Surgery Kaohsiung Medical University Hospital, Kaohsiung Medical University Kaohsiung Taiwan; ^2^ Department of Pathology Kaohsiung Medical University Hospital, Kaohsiung Medical University Kaohsiung Taiwan; ^3^ Department of Surgery, Faculty of Medicine College of Medicine, Kaohsiung Medical University Kaohsiung Taiwan

1

*BRAF* ^*V600E*^‐mutant metastatic colorectal cancer accounts for approximately 8%–10% of all colorectal malignancies worldwide and is associated with aggressive tumor biology, poor differentiation, and an unfavorable prognosis [1]. Mixed adenocarcinoma plus undifferentiated carcinoma is a rare histologic subtype that is also associated with poor clinical outcomes [2].

A 72‐year‐old man with a past medical history of laryngeal cancer presented with progressive abdominal fullness, lower back pain, and bilateral lower extremity edema. Abdominal computed tomography revealed a large retroperitoneal mass, initially suspected to represent lymphoma or retroperitoneal metastatic disease (Figure 1A). Subsequent colonoscopy revealed a non‐granular laterally spreading tumor in the descending colon, and biopsy confirmed moderately differentiated adenocarcinoma.

**FIGURE 1:**
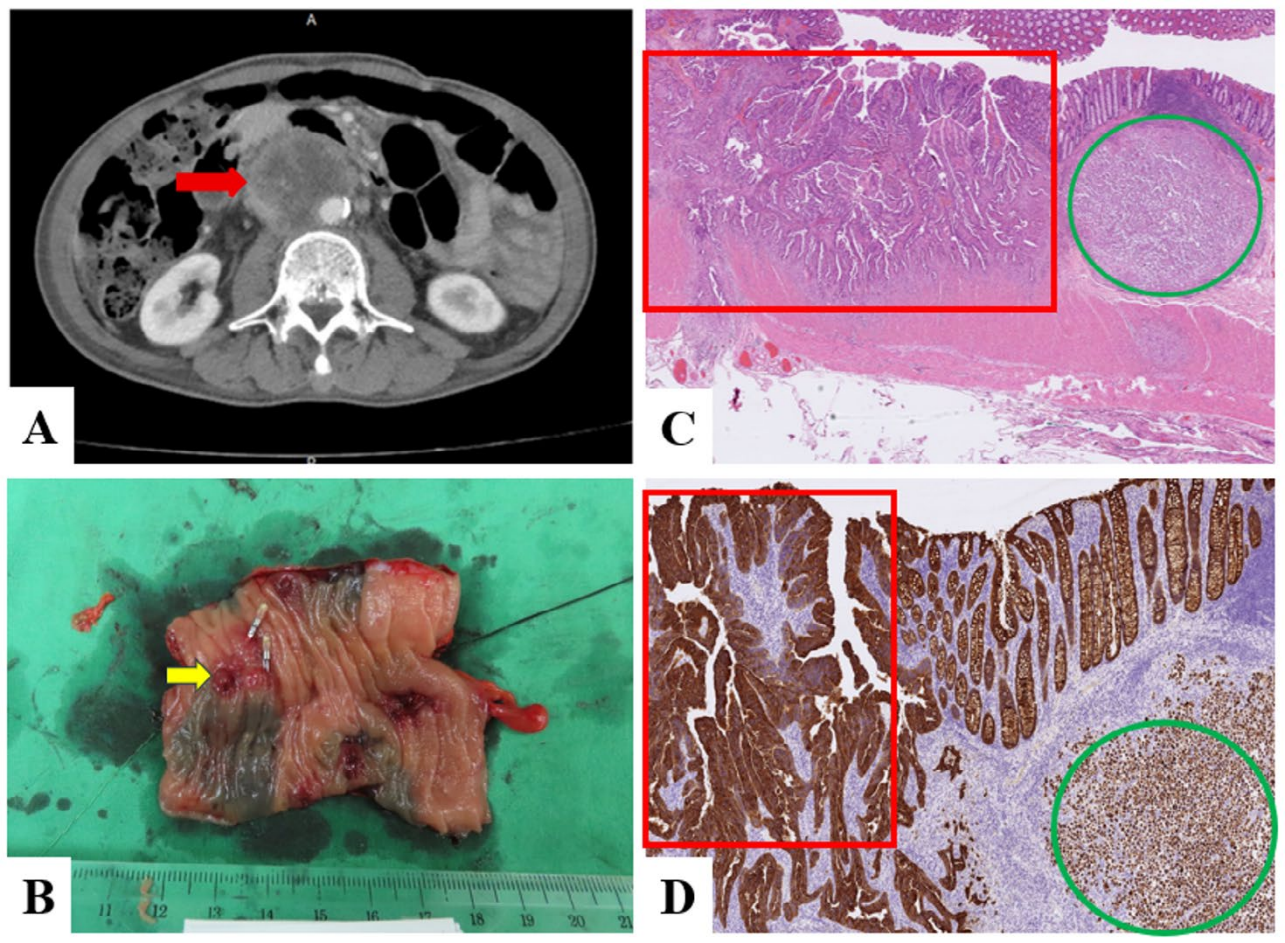
Mixed adenocarcinoma plus undifferentiated carcinoma of the descending colon with bulky retroperitoneal metastasis. (A) Axial abdominal computed tomography (CT) image presenting a large retroperitoneal mass (red arrow) causing vascular compression. (B) Gross left hemicolectomy specimen showing the primary descending colon tumor (yellow arrow). (C) Microscopic view indicating a mixed tumor composed of moderately differentiated adenocarcinoma (red box) and undifferentiated carcinoma (green circle area; hematoxylin–eosin). (D) Immunohistochemical staining indicating positivity for CK and CK20, focal positivity for SATB2, and negativity for CK7 and CDX2, supporting a colorectal primary origin (original magnification, ×40). The red box indicates the moderately differentiated adenocarcinoma component, whereas the green circle area indicates the undifferentiated carcinoma component.

The patient underwent left hemicolectomy with biopsy of the retroperitoneal mass (Figure 1B). Histopathological examination of the surgical specimen revealed a mixed tumor composed of adenocarcinoma (60%) and undifferentiated carcinoma (40%) (Figure 1C). Immunohistochemical staining was positive for pan‐cytokeratin and Cytokeratin 20 and focally positive for Special AT‐rich sequence‐binding protein 2 but negative for CK7 and CDX2. These findings supported a colorectal primary origin (Figure 1D). Pathological analysis of the retroperitoneal biopsy confirmed metastatic carcinoma. Comprehensive molecular profiling identified a *BRAF* ^*V600E*^ mutation with wild‐type *KRAS* and *NRAS*.

*BRAF* ^*V600E*^‐mutant metastatic colorectal cancer typically exhibits resistance to conventional cytotoxic chemotherapy and is associated with dismal overall survival [1, 3]. The landmark BEACON CRC trial established the combination of encorafenib and cetuximab as standard targeted therapy for pretreated patients [4]. Furthermore, the BREAKWATER trial demonstrated that combining encorafenib and cetuximab with mFOLFOX6 significantly improves clinical outcomes in the first‐line setting [5]. As per his molecular profiling results, our patient received systemic therapy with encorafenib, cetuximab, and chemotherapy.

In conclusion, this case describes an unusual presentation of a small primary descending colon cancer with mixed adenocarcinoma and undifferentiated carcinoma, manifesting as a bulky retroperitoneal metastasis. Early and comprehensive molecular profiling is crucial for accurate evaluation of prognosis and timely selection of effective targeted therapy in this aggressive subtype of colorectal cancer.

## Conflicts of Interest

The authors declare no conflicts of interest.

## Data Availability

The data that support the findings of this study are available from the corresponding author upon reasonable request.
